# Depressive symptoms in patients with epilepsy and clinically associated features in a single tertiary center

**DOI:** 10.1007/s10072-021-05589-1

**Published:** 2021-09-15

**Authors:** Mariacarolina Vacca, Mariana Fernandes, Matteo Spanetta, Fabio Placidi, Francesca Izzi, Caterina Lombardo, Nicola Biagio Mercuri, Claudio Liguori

**Affiliations:** 1 Department of Psychology, Sapienza University of Rome, Rome, Italy; 2 Department of Systems Medicine, University of Rome Tor Vergata, Rome, Italy; 3 Epilepsy Center, Neurology Unit, University Hospital of Rome Tor Vergata, Rome, Italy; 4 IRCSS Santa Lucia Foudantion, Rome, Italy

**Keywords:** Depressive symptoms, Generalized seizure, BDI-II; Comorbidity, Adulthood

## Abstract

Although depressive symptoms are the most common psychiatric comorbidity in epilepsy, they remain underestimated and untreated in a large proportion of patients. The purpose of this study was to evaluate depression severity and related clinical features in people with epilepsy using a well-reliable self-report index of mood, the Beck Depression Inventory-II (BDI-II). One-hundred seventeen adult patients with epilepsy were recruited from a tertiary epilepsy center and completed the BDI-II. A single-item analysis of the 21 questions of the BDI-II was computed and differences between women and men in each depressive symptom were evaluated. Correlation and regression analyses were used to identify clinical features associated with the severity of depression. Results showed gender differences in some items, with women reporting overall higher depression severity than men. The most common symptoms regarded domains of sleeping patterns, tiredness, and loss of energy. Regression evidence suggested that being female, having an epilepsy duration < 10 years, as well as being treated with psychotropic drugs and reporting generalized seizure, were associated with higher depression severity. Despite its cross-sectional nature, this study reinforces the importance of investigating and possibly treating depressive symptoms in adult patients with epilepsy, since they negatively impact well-being, daytime activities, and sleep. Further studies identifying pharmacological and non-pharmacological treatments for depression in epilepsy need to be planned.

## Introduction

Epilepsy affects all aged people and results in profound physical and psychopathological consequences [1–3]. Notably, comorbid psychiatric conditions are highly prevalent among patients with epilepsy as compared to the general population [2, 3] suggesting that psychiatric illnesses often accompany the diagnosis of epilepsy [4], or even precede the onset of the disease [5]. Moreover, the most common interictal psychiatric disease in epilepsy is depression [6]. Accordingly, individuals with epilepsy report significantly elevated odds of depression than those without epilepsy, even after adjusting for potential confounders (e.g., age, gender, comorbid physical illnesses [2]). Among the potential neurobiological determinants, epilepsy-related variables such as duration, frequency and severity of seizures, and medication have been of particular interest [7]. Therefore, previous investigations have attempted to evaluate the association between the aforementioned risk factors and depression in epilepsy.

Despite depression is a frequent psychiatric condition in individuals with epilepsy, its prevalence is often underestimated [8]. This evidence seems particularly alarming when considering that some studies reporting depression may exacerbate epilepsy-related symptoms (e.g., fatigue, stress, sleep problems) [9, 10] and hinder the positive outcomes of epilepsy treatment [11]. Systematic evidence suggests an overall prevalence of depression of 22.9–23.1% [12] and a lifetime prevalence for major depression ranging between 8 and 48% in patients with epilepsy [6]. Depressive symptoms in epilepsy affect patients’ well-being and the clinical course of the disease [13, 14]. The use of some anti-seizure medications (ASMs) to manage seizures can significantly affect mood by alleviating or exacerbating depressive symptoms [15]. On the other hand, some ASMs (e.g., GABAergic drugs) have been related to the worsening of mood symptoms in epilepsy [16, 17].

Predominantly, interest of clinicians focused on identifying valid assessment tools to robustly detect the presence of depressive symptoms among patients with epilepsy [18]. A recent systematic review on the validity of depression-screening tools in epilepsy reports that Beck Depression Inventory (BDI) [19] is a complete instrument to evaluate depressive symptoms in people with epilepsy for screening purposes, as compared to other self-report scales [20]. Studies employing this inventory, and, in particular, its later version (BDI-II) [21] found that the BDI total score often distinguished depressed from non-depressed participants with epilepsy [22, 23], confirming its well-recognized sensitivity. Some authors employ a single-item analysis approach in order to investigate the distribution of each specific facet assessed through the BDI and its relationship with epilepsy-related aspects [24]. The inspection of the single item provides valuable additional information on the core affective, somatic, and cognitive features of depression among individuals with epilepsy.

Hence, in this study, we investigated depressive symptoms in patients with epilepsy visited in a tertiary clinical center, by using the BDI-II with a single-item approach in order to detect the wide spectrum of depressive symptoms in this population of patients. Moreover, considering that gender may influence the prevalence of depression in epilepsy [25, 26], this study aims to compare depressive symptom patterns according to the BDI-II between female and male patients affected by epilepsy. Consistently with previous evidence [25], we expected that depressive symptoms would be more accentuated among female patients. Finally, we investigated the possible influence of seizure type, etiology of epilepsy, monthly total seizure count, and ASMs on depressive symptoms.

## Method

### Participants and procedure

Sample was collected during a 6-month period (from June 2020 to November 2020) with consecutive patients. Participants affected by epilepsy admitted to the Epilepsy Center of the University Hospital of Rome Tor Vergata and diagnosed according to the current clinical criteria suggested by the International League Against Epilepsy were invited to take part to the study. Upon acceptance, all patients were classified according to the recent classification of epilepsy and seizures by three experts in epilepsy (CL, FP, and FI) [27]. All patients underwent a routine neuropsychiatric evaluation involving a full neuropsychiatric history and anamnesis interview according to DSM-IV criteria [28] by a consulting neurologist. The study was approved by the Independent Ethical Committee of the University Hospital of Rome Tor Vergata and all patients gave their written informed consent to participate (R.S. 191/17 - 192/17; Eudract 2017-000990-35). The following demographic and clinical data were analyzed: age, sex, time since epilepsy onset, etiology, 1-month total seizure count, and concomitant ASMs. Inclusion criterion for this study was only age ≥ 18 years. Exclusion criteria were intellectual disability and/or presence of seizures the day of the outpatient visit.

### Instruments

The BDI-II [21, 29] was used to assess depressive symptoms and administered to each patient during a single session on an individual basis. It consists of 21 items with four possible responses, with higher total scores indicative of greater severity of symptoms. Scores ranging from 0 to 13 indicate minimal/no symptoms, 14–19 indicate mild depressive symptoms, 20–28 indicate moderate depressive symptoms, and 29–63 indicate severe depressive symptoms. Although these cut-off points have been provided for the general population, many authors have sustained their validity in detecting depression severity among patients with epilepsy [23]. In the present study, the reliability of BDI-II based on McDonald’s omega coefficient (ωt) [30] was high (ωt = .901) as well as Cronbach alpha coefficient (*α*= .898), as found in previous studies [31].

### Data analysis

#### Descriptive statistics and group comparison

First, descriptive statistics were computed to characterize the sample in terms of gender, age, epilepsy duration, epilepsy type, seizure frequency, seizure type, anti-seizure medication (ASM), and the use of antidepressants and antipsychotic drugs. The normality of the data was assessed through the Shapiro-Wilk test [32]. To minimize false-positive tests of significance, we set a significance level of *p* < .01 [33]. Mann-Whitney *U* tests were used to examine scores on each items of BDI-II, as did previous authors [34], and to assess the statistical significance of differences in BDI-II scores and item scores between female and male participants. Mann-Whitney *U* test is a rank-based procedure which is more appropriate with ordinal and not-normally distributed data. Gender comparison was also computed to examine differences in clinical characteristics.

#### Correlation and regression analyses

The Tau-Kendall correlation analysis was used to assess the statistical relationships among the variables included in this investigation. The Kendall correlation coefficient [35] is an appropriate measure of associations between categorical independent variables (e.g., seizure type) and continuous variables (e.g., BDI-II total score) [36]. Finally, a hierarchical linear regression analysis was computed in order to identify which demographic and clinical characteristic better predicted depressive symptoms severity. A two-stage hierarchical multiple regression was conducted with BDI-II scores as the dependent variable. Independent variables were analyzed separately in two blocks: age and gender in the first block, epilepsy duration, epilepsy type, seizure type, seizure frequency, number of ASM, and number of psychotropic drugs as predictors. Age and BDI total score were entered in the model as continuous variables, while epilepsy duration (< 12 months; 13 months–10 years; > 10 years), epilepsy type (structured, unknown), seizure type (unknown, focal, generalized), seizure frequency in the previous month (seizure-free, 1 episode, ≥ 2 episodes), and number of ASM (1–3) were coded as dummy variables. For this aim, the first category (i.e., epilepsy duration: < 12 months; seizure type: unknown; seizure frequency: seizure-free; number of ASM: 1) of each of the aforementioned ordinal variable was coded as the reference category. Since there was considerable heterogeneity in epilepsy etiology (see Table 1), the two major classifications (i.e., structured, unknown) were entered in the regression model.

**Table 1:** Descriptive characteristics of the sample

Characteristics	*N* (%)
*N* = 117	
**Age**^**a**^	M = 53.05 (±19.69)
< 30	18 (16.36)
30–40	15 (13.63)
41–50	20 (18.18)
51–60	13 (11.81)
> 60	44 (40)
**Sex**	
Males	56 (47.9)
Females	61 (52.1)
Epilepsy duration	
≤ 12 months	19 (17.8)
13 months–10 years	51 (47.7)
>10 years	37 (34.6)
**Epilepsy etiology**	
Structural	51 (46.4)
Unknown	44 (40)
Genetic	8 (7.3)
Autoimmune	2 (1.8)
Metabolic	2 (1.8)
Encephalopathic	2 (1.8)
Infective	1 (.9)
**Seizure type**	
Generalized	43 (39.1)
Focal	40 (36.4)
Mixed	27 (24.5)
**Seizure frequency**^**b**^	
Seizure-free	84 (76.4)
1 episode	17 (15.5)
≥ 2 episodes	9 (8.2)
**ASM**	
1	78 (70.9)
2–3	32 (29.1)
Psychotropic drugs	26 (23.6)
No psychotropic	84 (76.4)
drugs	

^a^Age represents age at time of entry into the data

^b^Seizure frequency in the last 28-day period before visit

Gender (1 = female; 2 = male) and the use of psychotropic drugs (0 = yes; 1 = no) were coded as dichotomic variables. Data were analyzed using the software program SPSS software version 25.0 [37].

## Results

### Description of the sample and prevalence rate of depressive symptoms in single-item analysis

A total of 117 participants aged 18–84 years were enrolled in the study. Nine patients (7.7%) did not manage to fill in questionnaires (language difficulties). Demographic and clinical characteristics are displayed in Table 1. Previous routine psychiatric evaluation indicated that five (4.3%) and four (3.4%) patients experienced anxiety and depression respectively, whereas for three patients (2.6%), signs of cognitive delay were observed. As regarding BDI-II scores, forty-two (38.8%) patients manifested depressive symptoms, with scores greater or equal to 14. Of those, 19 (17.6%) had mild symptoms (BDI-II scores: 14–19), 13 (12 %) reported moderate symptoms (BDI-II scores: 20–29), and the remaining 10 (9.3 %) patients had severe symptoms. At the question regarding suicide thoughts/attempts, 9 (8.3%) patients answered 1 (“I have thoughts of killing myself, but I would not carry them out”), and 8 (7.4%) answered 2 (“I would like to kill myself”).

The most frequent reported symptoms of depression were changes in sleeping patterns (*N* = 66; 61.1%), tiredness or fatigue (*N* = 66; 61.1%), and loss of energy (*N* = 66; 61.1%). Single-item response rates for female and male participants are displayed in Table 2.

**Table 2:** Items scores frequencies observed in the two groups and in the total sample

BDI-II items	Females (*n* = 57)			Males (*n*= 51)			Total (*n* = 108)		
	Score 0	Score 1	Score 2–3	Score 0	Score 1	Score 2–3	Score 0	Score 1	Score 2–3
	*n* (%)	*n* (%)	*n* (%)	*n* (%)	*n* (%)	*n* (%)	*n* (%)	*n* (%)	*n* (%)
1. Sadness	29	21	7	40	11	0	69	32	7
	(50.9%)	(36.8%)	(12.3%)	(78.4%)	(21.6%)	(0%)	(63.9%)	(29.6%)	(6.5%)
2.Pessimism	28	15	14	32	14	5	60	29	19
	(49.1%)	(26.3%)	(24.6%)	(62.7%)	(27.5%)	(9.8%)	(55.6%)	(26.9%)	(17.6%)
3.Past failure	35	9 (15.8%)	13 (22.8%)	42	6 (11.8%)	3	77	15	16
	(61.4%)			(82.4%)		(5.9%)	(71.3%)	(13.9%)	(14.8)
4.Loss of pleasure	25	18	14 (24.6%)	31	17	3	56	35	17
	(43.9%)	(31.6%)		(60.8%)	(33.3%)	(5.9%)	(51.9%)	(32.4%)	(15.8%)
5.Guilty feelings	31	21	5	40	11	0	71	32	5
	(54.4%)	(36.8%)	(8.8%)	(78.4%)	(21.6%)	(0%)	(65.7%)	(29.6%)	(4.6%)
6.Punishment feelings	38	8	11	42	4	5	80	12	16
	(66.7%)	(14%)	(19.3%)	(82.4%)	(7.8%)	(9.8%)	(74.1%)	(11.1%)	(14.8%)
7.Self-dislike	37	15	5	42	9	0	79	24	5
	(64.9%)	(26.3%)	(8.8%)	(82.4%)	(17.6%)	(0%)	(67.5%)	(22.2%)	(4.6%)
8.Self-criticalness	38	9	10	38	5	8	76	14	18
	(66.7%)	(15.8%)	(17.6%)	(74.5%)	(9.8%)	(15.7%)	(70.4%)	(13%)	(16.7%)
9.Suicidal thoughts/wishes	46	6	5	45	3	3	91	9	8
	(80.7%)	(10.5%)	(8.8%)	(88.2%)	(5.9%)	(5.9%)	(84.3%)	(8.3%)	(7.4%)
10.Crying	25	10	22	44	3	4	69	13	26
	(43.9%)	(17.5%)	(38.6%)	(86.3%)	(5.9%)	(7.8%)	(63.9%)	(11.1%)	(24%)
11.Agitation	24	21	12	31	19	1	55	40	13
	(42.1%)	(36.8%)	(21%)	(60.8%)	(37.3%)	(11.1%)	(50.9%)	(37%)	(12%)
12.Loss of interest	33	16	8	33	15	3	66	31	11
	(57.9%)	(28.1%)	(5.9%)	(64.7%)	(29.4%)	(5.9%)	(61.1%)	(28.7%)	(10.2%)
13.Indecisiveness	30	13	13	30	15	5	60	28	18
	(53.6%)	(23.2%)	(23.2%)	(60%)	(30%)	(10%)	(56.6%)	(26.4%)	(17%)
14.Worthlessness	37	13	5	39	7	3	76	20	8
	(67.3%)	(23.6%)	(9.1%)	(79.6%)	(14.3%)	(6.1%)	(73.1%)	(19.2%)	(7.6%)
15.Loss of energy	16	21	19	24	19	7	40	40	26
	(28.6%)	(37.5%)	(33.9%)	(48%)	(38%)	(14%)	(37.7%)	(37.7%)	(24.6%)
16.Changes in sleeping pattern	14	24	17	25	21	4	39	45	21
	(25.5%)	(43.6%)	(30.9%)	(50%)	(42%)	(8%)	(37.1%)	(42.5%)	(20%)
17.Irritability	23	23	10	29	18	3	52	41	13
	(41.1%)	(41.1%)	(17.9%)	(58%)	(36%)	(6%)	(49.1%)	(38.7%)	(12.3%)
18.Changes in appetite	29	16	11	32	15	3	61	31	14
	(51.8%)	(28.6%)	(19.6%)	(64%)	(30%)	(6%)	(57.5%)	(29.2%)	(13.2%)
19.Concentration difficulty	20	20	15	24	20	6	44	40	21
	(36.4%)	(36.4%)	(27.3%)	(48%)	(40%)	(12%)	(41.9%)	(38.1%)	(20%)
20.Tiredness or fatigue	14	23	19	26	22	2	40	45	21
	(25%)	(41.1%)	(33.9%)	(52%)	(44%)	(4%)	(38.1%)	(42.5%)	(20%)
21.Suicidal thoughts or wishes	30	10	13	40	6	4	70	16	17
	(56.6%)	(18.9%)	(23.2%)	(80%)	(12%)	(8%)	(68%)	(15.5%)	(16.5%)

### Gender differences

Analysis on group comparison showed no significant differences between females and males on epilepsy-related characteristics (see Table 3).

**Table 3:** Mann-Whitney *U* test. Gender comparison on epilepsy-related variables

Variable	*U*	*p*
Epilepsy duration	1014.500	.212
Epilepsy type	1172.500	.922
Seizure type	1125.500	.212
Seizure number	1105.000	.723
ASM	1076.500	.431
Psychotropic drugs	1223.000	.115

**Fig. 1:**
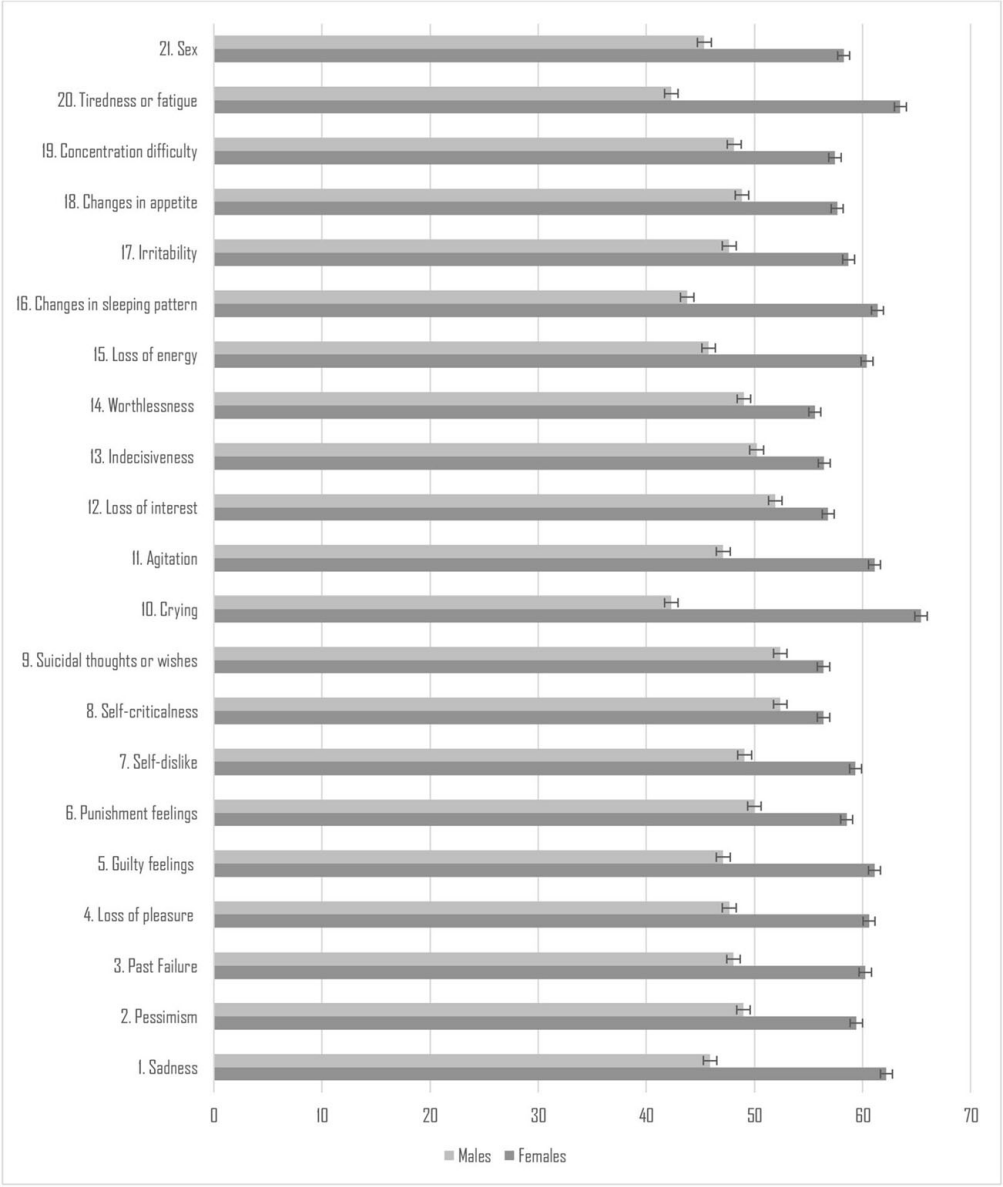
Mean rank differences of each BDI-II item score between males and females

Results on depressive symptoms showed a significant gender difference in BDI-II total score (*U* = 791.5, *p* = 000), with females reporting higher scores (mean rank = 65.55) than males (mean rank = 41.15). Compared with males, females rated the following items significantly higher: sadness (*U* = 1014.500, *p* = .001), guilty feelings (*U* = 1076.500, *p* = .005), crying (*U* = 832.000, *p* = .000), loss of energy (*U* = 1013.000, *p* = .009), changes in sleeping pattern (*U* = 914.000, *p* = .001), tiredness or fatigue (*U* = 840.500, *p* = .000), loss of interest in sex (*U* = 993.000, *p* = .008), and agitation (*U* = 1076.500, *p* = .010) (Figure 1).

The result on item 16 (changes in sleeping pattern) revealed that, overall, females reported more problematic sleep pattern as compared to males. The analysis of more frequent reported symptom about sleep impairment revealed that 29.5% (*n* = 18) of females reported to sleep more than usual and that another same amount reported to sleep less than usual. Different results were observed in the male sample: 25% (*n* = 14) showed to sleep less than usual and 16.1% (*n* = 9) reported to sleep more than usual.

### The association between demographic and clinical characteristics and BDI-II score

#### Correlation analysis

Results of Kendall correlation analysis showed significant and positive associations between age and generalized seizure, focal seizure, the presence of structured epilepsy, and the use of psychotropic drugs (*p* < .01). Moreover, a negative correlation of age with unknown etiology of epilepsy was observed, suggesting that younger patients did not present evident causes of it. Epilepsy duration was correlated with the incremental use of ASM (*p* < .01), whereas BDI-II score was associated with female gender and the use of psychotropic drugs (*p* < .01).

#### Regression analysis

The collinearity statistics (i.e., Tolerance and VIF) were all within acceptable limits [38], and an examination of the Mahalanobis distance scores [39] indicated no multivariate outliers. The hierarchical multiple regression revealed that the first step accounted for 13.2% of the variation in BDI-II, with gender contributing significantly to the regression model, *F* (2,98) = 7.439, *p* < .01. At the second step, the introduction of clinical characteristics explained an additional 25.1% of variation in BDI-II, and this change in R2 was significant, *F* (13,87) = 4.155, *p* < .001.

A significant and negative regression coefficient was observed for epilepsy duration > 10 years, indicating that longer epilepsy duration was associated with a lower BDI-II score. On the other hand, the use of psychotropic drugs and generalized seizures were independent positive determinants of depressive symptoms.

Moreover, a negative regression coefficient was observed for gender, indicating that females showed a trend towards being significantly associated with the BDI-II index. The predicted multiple regression model accounted jointly for 38.3% of the variation of the BDI-II (Table 4).

**Table 4:** Summary of hierarchical regression analysis for variables predicting BDI-II total score

Variable	*β*	*t*	sr^2^	*R*	*R*^*2*^	*∆R*^*2*^
**Step 1**				.363	.132	.132
Gender	−.366	-3.821	−.360			
Age	−.020	−.211	−.020			
**Step 2**				.619	.383	.251
Gender	−.333	−3.761^***^	−.317			
Age	−.081	−.835	−.070			
*Seizure frequency*						
One episode	−.054	−.604	−.051			
≥ 2 episodes	.086	.904	.076			
*Number of ASM*						
ASM = 2	−.044	−.500	−.042			
ASM =3	.173	1.834	.154			
*Epilepsy duration*						
13 months–10 years	−.197	−1.597	−.134			
> 10 years	−.349	−2.694^**^	−.227			
*Epilepsy type*						
Structured	.040	.264	.022			
Unknown	.130	.909	.077			
*Seizure type*						
Generalized	.228	2.100^*^	.177			
Focal	−.055	−.498	−.042			
Psychotropic drugs	.347	3.831^***^	.323			

Note: ^*^p < .05, ^**^p < .01, ^***^p< .001

## Discussion

The first aim of the present study was to examine the presence of depressive symptoms in patients with epilepsy visited in a tertiary clinical center, by using the BDI-II with a single-item analysis. This approach is appropriate to examine the distribution of individual item of the BDI-II providing additional information on the core features of depression among individuals with epilepsy [24].

Results showed that around 39% of participants reported clinically defined depressive symptoms, suggesting that an association between depression and epilepsy should be inferred. This finding is consistent with previous studies reporting similar percentage rates in Italian samples [23] and with the evidence on the frequency of depression in patients with epilepsy ranging from 9 to 37% [40]. Indeed, this well-known variability of depressive symptoms reported by patients with epilepsy might be due to the heterogeneity of tools and methods employed to screen for depression in the published literature (e.g., self-reported scales, psychiatric interview; [41]). This variety often makes comparisons between studies impossible [40] thus being a barrier to understanding the relationship between depression and epilepsy.

The underling mechanisms explaining the epilepsy–depression link highlight the contribution of both biological and psychosocial factors [42]. More specifically, the biological determinants of this association (e.g., hippocampal shrinking, amygdala hypertrophy) have been observed in neuroimaging and neurobiological studies and, similarly, the implication of psychological variables (e.g., stigmatization, coping mechanism) in the pathogenesis of depression in epilepsy has been also reported [43]. Epidemiological studies sustain a bidirectional relationship between epilepsy and depression, as depression may not only co-occur with and follow the onset of epilepsy [5] but also may increase the risk of developing this disease [44]. Overall, this evidence seems to suggest an underlying common neurobiological basis [45].

Results of the current study confirmed what was found in previous evidence [25], suggesting that depressive symptoms are more accentuated among female patients than male patients presenting epilepsy. Evidence on the general population suggests part of the gender gap might be explained by heightened exposure to severe adversity and by structural social gender inequity [46]. Consistently, gender was a significant and unique predictor of high BDI-II scores, above and beyond the clinical features of the sample. This evidence was consistent with results from gender comparison and reinforces the idea that being female is a strong depression-predisposing factor in epileptic patients [47]. A recent meta-analysis highlights that the female hormonal environment could be one explanation of this phenomenon, as the decrease in estrogen increases the likelihood of depression [8]. Furthermore, females typically face with challenging conditions that may be affected by epilepsy and significantly impact on their perceived quality of life, resulting in high negative emotionality. For example, an increased prevalence of depression during and after pregnancy in women with epilepsy as compared to women with other chronic diseases and women without epilepsy has been observed [47]. Also, some authors suggest that adverse effects of epilepsy (e.g., stigma, discrimination, vocational difficulties) may prevent the achievement of a job and cause depression in women [48]. Moreover, females may be more prone to disclosing depressive symptoms than men [8]. Therefore, future studies should assess factors possibly explaining the gender role in the depression-epilepsy association, and longitudinal research across the lifespan, examining risk factors and transdiagnostic outcomes, is needed.

The present study also examined the associations between depressive symptoms and demographic and clinical characteristics in patients with epilepsy. Differently from previous authors [49, 50] who found that a shorter epilepsy duration was significantly associated with a lower risk of depression, in the present investigation, high epilepsy duration was determined to decrease depression. This result is consistent with a previous study that found a negative correlation between epilepsy duration and BDI-II scores in patients with temporal lobe epilepsy [51]. A possible explanation for this rather contradictory result is that adults with long-term epilepsy “come to terms with their ailment over time” [52 , p. 63]. In this context, it is noteworthy that a previous analysis of 99 adults suffering from intractable epilepsy found an association between longer duration of epilepsy and higher quality of life scores [53]. A long-term epilepsy may reflect a greater span of time available to face stressful difficulties brought about by this disease. Future studies on the current topic are therefore recommended in order to elucidate if perceived difficulties in coping with epilepsy-related disabilities (e.g., loss of independence) are associated with the duration of symptoms.

The positive unique association found in the present study between the use of psychotropic drugs and depression may indicate that patients with high severity of depression already received psychiatric medications. This finding reinforces the clinical supposition that psychiatric medications cannot present the same effectiveness in patients with epilepsy, thus justifying the need of personalized approaches, counting both pharmacological and non-pharmacological treatments, in patients with epilepsy taking into account that the disease per se can alter the neurobiological networks at the basis of depressive symptoms. Notably, the evidence that, among patients reporting depressive symptoms according to the BDI-II score, almost half (i.e., 57%) did not already receive psychotropic medications might suggest that, in many cases, patients screened as positive for depression with this instrument are under-treated, consistently with previous observations [54]. This fact is not entirely unfavorable, considering the high rate of false positives for depression associated with the use of the BDI-II [55]. Conversely, it appeared that depressive symptoms can be overlooked in patients with epilepsy, in particular when the patients are seizure free. Further investigations should comply with the recommendation that the diagnosis of depression and the relative need of a treatment plan must be confirmed with a clinical interview conducted by a trained clinician [56].

The lack of associations with other epilepsy-related variables confirmed previous studies evidencing no significant changes in epilepsy characteristics (e.g., seizure frequency) in patients treated with antidepressants [57]. More specifically, in the present study, the seizure frequency was not associated with the severity of depression as indicated by regression coefficients. This result is confirmed by the evidence that the number of seizure-free patients in the current sample was very high (i.e., 76%), thus suggesting that the high prevalence of depressive symptoms found (39%) can only be partially explained by factors related to epilepsy. Previous authors suggested that epilepsy per se is related to the severity of depression, thus encouraging clinicians to treat all patients for depression irrespective of seizure frequency [54].

In the current sample, the presence of generalized seizure significantly predicted severity of depression. This result did not appear to corroborate previous evidence demonstrating that, in comparison to generalized seizures, focal seizures were associated with a higher risk of depressive symptoms [58]. On the other hand, this evidence substantiates previous findings in the literature indicating that patients diagnosed with generalized seizure are the most vulnerable to severe depression [59, 60].

The novelty of this study relies in the single-item analysis documenting that, among all the depressive symptoms, one of the most pronounced was changes in sleeping patterns, confirming that disrupted sleep patterns in epilepsy are commonly described clinically [61]. High percentage of patients also reported tiredness and loss of energy, suggesting that patients with epilepsy frequently experience extreme and persistent weakness or exhaustion [62]. Moreover, the high prevalence of all these symptoms may corroborate previous systematic evidence that sleep-related problems and fatigue are intercorrelated symptoms of depression in epileptic condition [9]. Future studies should extend the above single-item analysis to examine depressive symptoms in specific epilepsy type and in subgroups of patients (e.g., pediatric). Finally, the rate of suicide ideation (i.e., item 9) observed in the present sample (15.7%) supported the prevalence of suicidal attempts found in previous evidence [63]. This finding encourages the development of appropriate suicide prevention strategies in conjunction with psychiatry and pharmacotherapy programs, especially considering that suicide is frequently underdiagnosed by physicians [64]. On the other hand, results concerning the depressive symptom rate in epilepsy should be interpreted with caution, since anergy, fatigue, and sleep disturbances are common symptoms reported by patients with epilepsy even in the absence of psychiatric conditions [9].

Some limitations are acknowledged in the present study including its cross-sectional nature, which does not allow demonstration of causal relationship between predictors and BDI-II scores. Future studies should address this issue, especially considering that no clear results on a directional association exist. Some studies supported the relationship between depression symptom and epilepsy-related symptoms (e.g., seizure frequency) to be bidirectional [65]. On the other hand, depression appeared to be a prospective predictor for seizure frequency [66]. Further prospective studies are needed to fill this gap. Another limitation was the relatively small sample size, which might had affected the conclusions of the study in terms of their generalizability. Moreover, the application of subjective self-report instruments to assess depressive symptoms did not permit the exclusion of social desirability in the responses given by participants. Finally, the lack of a control group enabled the comparison between patients with epilepsy and healthy participants on depressive scores.

## Conclusions

Despite the aforementioned limitations, the current study strengthens the imperative to screen for depression in epilepsy condition. Results suggested that female gender, short epilepsy duration, the use of psychotropic drugs, and generalized seizure were significantly associated with the severity of depression. Evidence on prevention and early diagnosis are still lacking [67, 68] and future treatment protocols focused on depression in epilepsy condition should account for the major trending observed in women.

### Author contribution

All authors contributed to the study conception and design. Material preparation and data collection were performed by Claudio Liguori, and analysis was performed by Mariacarolina Vacca. The first draft of the manuscript was written by Mariacarolina Vacca and all authors commented on previous versions of the manuscript. All authors read and approved the final manuscript.

## Data Availability

The datasets generated during and/or analyzed during the current study are available from the corresponding author on reasonable request.
